# Seasonal and inter-annual  variation  in exposure to peregrines (*Falco peregrinus)* for southbound western sandpipers (*Calidris mauri*)

**DOI:** 10.1186/s40462-022-00343-4

**Published:** 2022-10-27

**Authors:** Ronald C. Ydenberg

**Affiliations:** Centre for Wildlife Ecology, Department of Biological Sciences, Simon Fraser University, Burnaby, Canada

**Keywords:** Predation danger, Long-distance migration, Anti-predator behavior

## Abstract

**Background:**

The western sandpiper (*Calidris mauri*) is an early southbound migrant species in North America. The ‘peregrine avoidance’ hypothesis proposes that this timing evolved to reduce exposure to their main predator, the peregrine (*Falco peregrinus*), along the Pacific flyway.

**Methods:**

I evaluate this hypothesis based on 16 years of near-daily (June – October) measures of peregrine presence made on the Fraser River estuary, a major stopover in the Pacific northwest.

**Results:**

Exposure to peregrines is lowest for the earliest southbound western sandpipers, and rises steeply as peregrines en route from northern breeding areas begin to arrive in late July or August. Peregrine arrival timing varies greatly between years, shifting in step with the onset of spring along coastal Alaska. Peregrine presence on the Fraser estuary on any date is higher in years with earlier spring onset. On the median adult sandpiper passage date (day-of-year 198) this increases 17-fold over the inter-annual range between the earliest and latest peregrine arrival dates.

**Conclusion:**

The pattern of strong and predictable changes in the seasonal pattern of danger quantified here provides a further test of the hypothesis that danger affects migratory timing. Western sandpipers appear to anticipate the exposure level of southward migration, perhaps because they are able to observe spring onset on their Alaskan breeding grounds. They adjust the duration of parental care and length of the breeding season to keep the date of migratory departure from the Arctic relatively invariant in spite of large interannual variation in spring onset. While underway they also adjust aspects of migratory behavior. These observations support the ‘peregrine avoidance’ hypothesis, and suggest that western sandpipers are able to counter, at least partially, the higher migratory danger of early spring years.

## Introduction

The timing of avian migration is widely believed to be influenced by peaks in food abundance at stopover sites [1, 2]. Many papers, for example, describe goose migration as surfing a ‘green wave’ of spring-induced grass growth [3]. Seaduck species may follow a ‘silver wave’ of herring spawning events along the west coast of North America [4]. Red knots *Calidris canutus rufa* migrating northwards along the American Atlantic coastline time their arrival in Delaware Bay to forage on horseshoe crab *Limulus polyphemus* eggs, abundant for only a short period [5]. Schneider & Harrington [6] suggest that southward migration timing of semipalmated sandpipers (*Calidris pusilla*) evolved to take advantage of temporally-restricted high prey abundance at stopover locations along migratory routes. And climate-change induced shifts in the timing of peak food availability are thought to select for parallel changes in the phenology of long-distance migrants - though some species are unable to shift rapidly enough to keep up [7].

Safety is also a key element of stopover site quality for migrants [8–10], but has received less attention than has food availability [11]. In some cases at least, migratory timing appears influenced by predation danger. Lehikoinen [12], for example, reported that the Eurasian sparrowhawk (*Accipiter nisus*), the main predator of passerines, advanced its autumn phenology by about ten days between 1979 and 2008, which he attributed to climate change. Over the same period, early-migrating passerines advanced while late-migrating species delayed passage. Both groups thereby reduced the overlap with peak sparrowhawk passage.

In a recent review Sabal et al. [13] describe interactions between migratory animals and their predators in a variety of taxa. They reviewed what is known of how predators affect the ecology and evolution of migration, and the means whereby migrants may mitigate the danger. They considered in particular human influences on this interaction, and how this knowledge might be applied to conservation.

The western sandpiper (*Calidris mauri*) is one of the earliest southward long-distance migrants in North America. The first adults are underway by the summer solstice, departing their Arctic breeding grounds while there are 24 h of daylight, often deserting young after relatively little parental care. Lank et al. [14, see also 15] propose that these traits evolved to enable adults to undertake southward migration before the appearance of predators along the flyway. Here I consider the timing of southbound migration of western sandpipers in relation to that of their most important predator, the peregrine falcon (*Falco peregrinus*), aiming to evaluate this hypothesis by developing a quantitative measure of sandpiper migratory exposure to peregrines.

The historical number of breeding pairs of peregrines in North America is estimated at 10,600–12,000, the majority of which (~ 75%) bred north of 55° in Arctic and boreal regions, including Greenland [16, p. 6]. Numbers were reduced to a few hundred breeding pairs during the early 1970s, subsequently began to rise after DDT was banned, and based on mid-winter counts along Pacific flyway are now well into substantial recovery [17]. After breeding, northern peregrines migrate on a broad front across North America, most to non-breeding sites spread across lower temperate and tropical coastlines of the Americas. This movement generates a transient zone of high danger with distinctive geography and phenology, progressing southward at ~ 200 km d^− 1^ [18]. This zone is an important feature of the continental-scale predator landscape for sandpipers [14] and other migrant species [11].

Western sandpipers proceed northward on their lengthy (~ 5–11,000 km) migration on a set schedule [19], arriving on Alaskan breeding areas shortly after the mean date of snowmelt (day-of-year 120, [20]). Southward migration is initiated quickly after breeding with a long crossing of the Gulf of Alaska to the Pacific northwest. This jump largely bypasses the breeding range of the ‘marine’ peregrine sub-species *pealei* [21], which breeds near seabird colonies. These peregrines as well as members of the sub-species *tundrius* and *anatum* (mostly Arctic and boreal region breeders) migrate or wander in late summer and autumn along the Pacific flyway (e.g. [22]), taking up non-breeding residence at sites as far as South America. Western sandpipers begin to encounter these peregrines upon their migratory arrival in the Pacific northwest.

Lank et al. [14] describe the annual pattern of peregrine presence on the Fraser River estuary in southwestern British Columbia, a major stopover site for southbound western sandpipers. The presence of peregrines here is low from May into July [14, see also 23], and rises steeply in late July or August to a plateau during October before declining through the winter. The steep summer increase is due to the post-breeding arrival of peregrines en route from northern breeding areas. Studies of western sandpipers (and its close relative the semipalmated sandpiper *Calidris pusilla*) document various adjustments made to increase migratory safety. These include the choice of feeding [9] and stopover sites [10], fuel load size [27], breeding behavior [25], wing morphology [35], as well as migratory speed [32] and routing [11], all of which have changed as peregrine numbers have risen since the mid-1970s. Here we test the hypothesis of Lank et al. [14] that early southward migration also evolved to increase migratory safety.

The timing of the steep summer increase in peregrine presence shifts in step with the onset of spring along coastal Alaska [20, see their Fig. 1], which is itself set by highly variable large-scale climate conditions in the northeast Pacific and adjacent continental areas [24]. For example, based on snowmelt on the Yukon-Kuskwomin delta, spring onset varied between years by 43 days in the 23-year (1978‒2000) data set assembled by Niehaus & Ydenberg [20] and peregrine passage varied positively with it by 54 days. ‘Spring’ varied by 28 days in the 3-year (2004–2006) field study by Jamieson et al. ([25]; spring based on ‘ice-out’ in the Kuyungsik River).

**Fig. 1:**
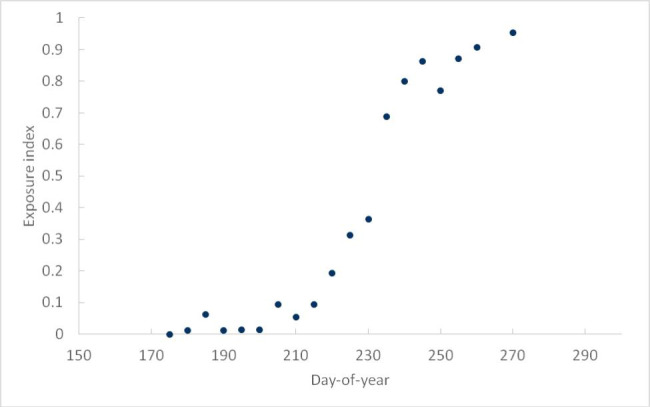
Seasonal presence of peregrine falcons on the Fraser estuary, based on ~ 1100 near-daily standardized 1 h surveys late June – late October, 1986–2002. The surveys are reported in Lank et al. (2003). A total of 1250 peregrine sightings were recorded (overall average 1.1 per survey). Day-of-year 175 is June 24; day-of-year 275 is October 2. Shown is the number of peregrines sighted daily, averaged over 5-day blocks centred on each point, and expressed as a proportion of the level reached during October of the same year. Note that the curve is not cumulative: it represents the proportional number of daily peregrine sightings. Note also that the peregrine arrival dates observed in this study are shown in Fig. 2

The migratory phenology of western sandpipers is far less variable. Their arrival in Alaska (d-o-y 125; [20]; this timing changed little 1985–2016; [26]) occurs just after the mean date of snowmelt. Breeding commences immediately if the tundra is snow-free, but otherwise they must wait for snowmelt. The interval between nesting and departure shortens when spring onset is earlier, and southward migration timing is hence relatively invariant [20]. The advanced phenology of falcons in early spring years thus results in greater migratory overlap with western sandpipers. In this paper I develop a quantitative measure of this effect.

## Methods

The analysis developed here is based on western sandpiper and peregrine migration phenology data reported in Lank et al. [14], and Niehaus & Ydenberg [20]. The reference location is the Fraser River estuary in south-west British Columbia. During northward passage, western sandpipers stopover on just a few large sites on the estuary, and are seen in greatest numbers (~ 10–50,000 daily) for two or three weeks from mid-April, with a marked peak in the last days of the month [26].

The number of sandpipers present each day during southward migration is lower by two or even three orders of magnitude than during northward migration. The reasons are that southward passage has a much longer duration, and many more stopover sites in the region are used than on northward passage [8]. Southbound migrants flow through steadily for about two months, with adults passaging during July (50% passage reached on average on d-o-y 198) and juveniles during August (50% passage reached on average d-o-y 228). Peregrine presence is also higher, suggesting that (if measured as the daily ratio between peregrines and western sandpipers) southbound migration is the more dangerous of the two.

Peregrine presence was measured at the Reifel Island Migratory Bird Sanctuary on the Fraser River estuary, located adjacent to large tidal mudflats used by migrant sandpipers [8]. Data are derived from ~ 1100 near-daily standardized 1 h surveys made June – October, 1986–2002, described in Lank et al. ([14]; see their Fig. 3). A total of 1250 peregrine sightings were recorded (overall average 1.1 per survey). The number of peregrines sighted annually climbed steadily through these years, a feature that has been discussed elsewhere [17, 27]. This paper focusses on the seasonal pattern.

**Fig. 2:**
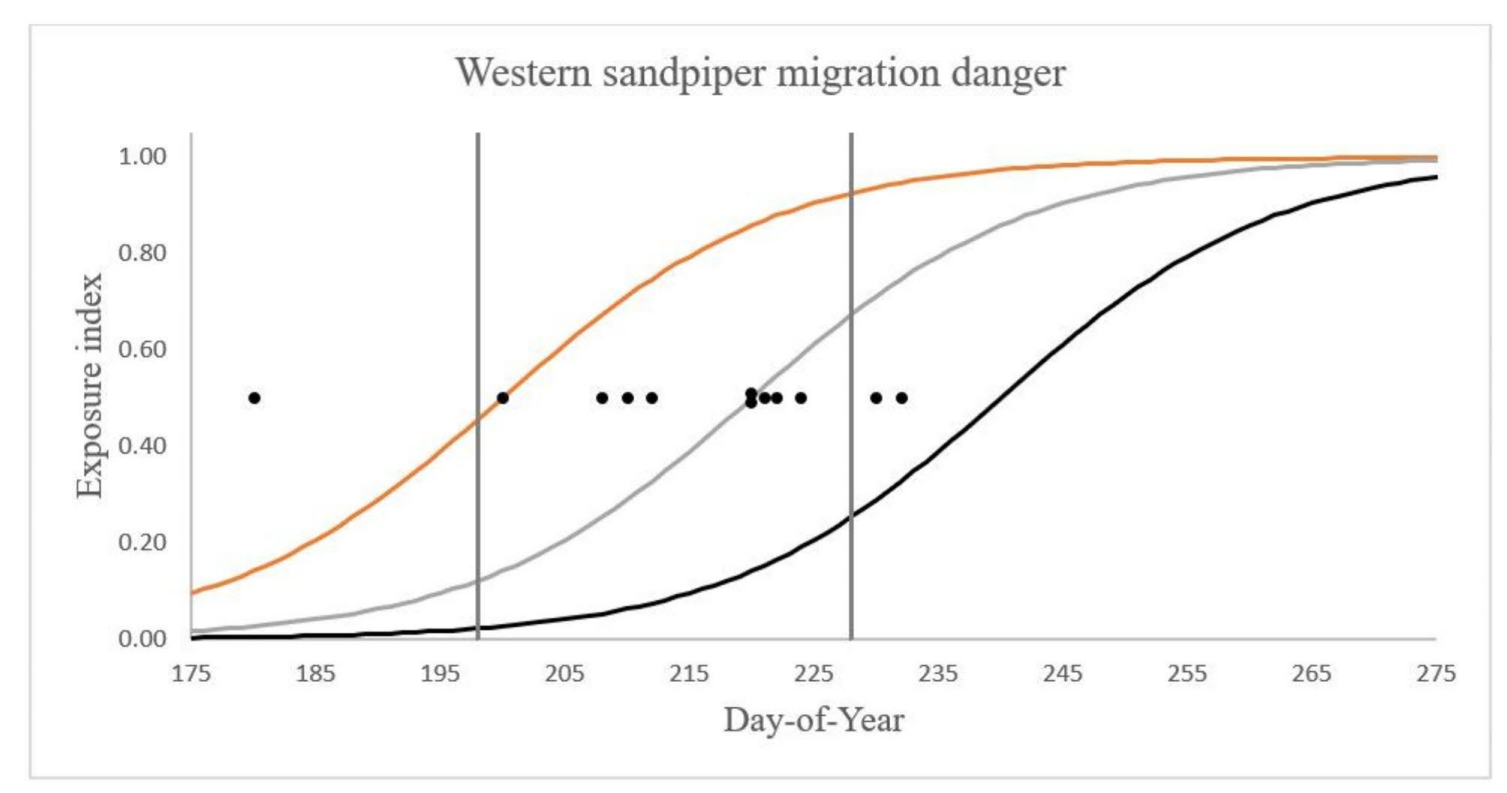
Annual progression of the exposure index (relative number of peregrines sighted daily) on the Fraser estuary, in years with varying peregrine arrival date (x-axis; *m* in Eq. 1). Peregrine arrival is defined as the date that 50% of the October level in that year is reached, with dots indicating the arrival dates observed in this study. The logistic curves used here are based on Fig. 1, shifted left or right to represent years with earlier or later peregrine arrival, respectively. Three examples are shown (orange: *m* = 200; grey: *m* = 220; black: *m* = 240). The mean passage dates for adult western sandpipers (day-of-year 198) and juveniles (day-of-year 228) are indicated by vertical lines

The daily record of peregrine sightings was divided into 5-day blocks between day-of-year 175 (June 24) and day-of-year 274 (October 1). The total number sighted in each block was tallied, and the daily average expressed as a proportion of the level reached during the month of October of the same year. This is termed the ‘exposure index’. The average over all 16 years of the record is shown in Fig. 1.

I defined peregrine ‘migratory arrival date’ on the Fraser estuary in each year as the day-of-year on which the exposure index reached 0.50 (from [20]). I fitted a logistic curve of the form.

1 $${\rm{d}}\left( {{\rm{t,m}}} \right){\rm{  =  L/(1  +  }}{{\rm{e}}^{\left( { - {k^*}\left( {t{\rm{ }}-{\rm{ }}m} \right)} \right)}})$$

to the points in Fig. 1. Here d(*t,m*) is the exposure index value on day-of-year *t* in a year with peregrine arrival date *m*. L is the asymptotic value (set to 1.0), and *k* is the logistic growth rate. Note that d(*t,m*) = 0.5 when *t = m*. The parameter *k* was adjusted to maximize the goodness-of-fit, assessed as the proportional reduction in the total sum of squares.

## Results

The average peregrine arrival date in the 16-year record is d-o-y 220 (August 8). As mentioned above, the arrival date varies widely between years, ranging from d-o-y 178 to 232, shifting in step with the onset date of spring on Alaskan breeding areas. Peregrine arrival on the Fraser estuary occurs on average 100.2 ± 5.3 (95% CI) days after the Alaskan spring onset date, and is later by 1.1 days for each day that snowmelt is later [20].

The seasonal progression of the exposure index, averaged over all 16 years of the record, is shown in Fig. 1, and is described by the logistic equation.

2 $${\rm{d}}\left( {t,m} \right){\rm{ }} = {\rm{ }}1/(1{\rm{ }} + {\rm{ }}{{\rm{e}}^{\left( { - {{0.09}^*}{\rm{ }}\left( {t{\rm{ }}-{\rm{ }}m} \right)} \right)}}$$

in which d(*t,m*) is the exposure index value on day-of-year *t* in a year with peregrine arrival date *m*. Equation (2) provides a good fit to the data in Fig. 1 (*m* = 220; r^2^ = 0.96), and facilitates comparisons between years by enabling the exposure index to be estimated in relation to the date of peregrine arrival on the Fraser estuary in the same year. This effect is modelled by using lower (higher) values of *m* to shift the curve leftward (rightward), representing earlier (later) peregrine arrival years.

The results are summarized in Fig. 2. Within each year, the exposure index rises during the southward passage period. This seasonal increase in peregrine presence was previously noted by Lank et al. ([14]; see their Fig. 3). However, the date-specific value in any year also depends strongly on the peregrine arrival date in that year. With the average peregrine arrival date (*m* = 220), the exposure index on the median adult sandpiper passage date (*t* = 198) is 0.12, much lower than that experienced by a juvenile (0.67) on its median passage date a month later (*t* = 228). In a year when *m* = 210 (10 days earlier than average), the exposure index on d-o-y 198 becomes 0.25 in place of 0.12, and on d-o-y 228 is increased to 0.83 from 0.67.

**Fig. 3:**
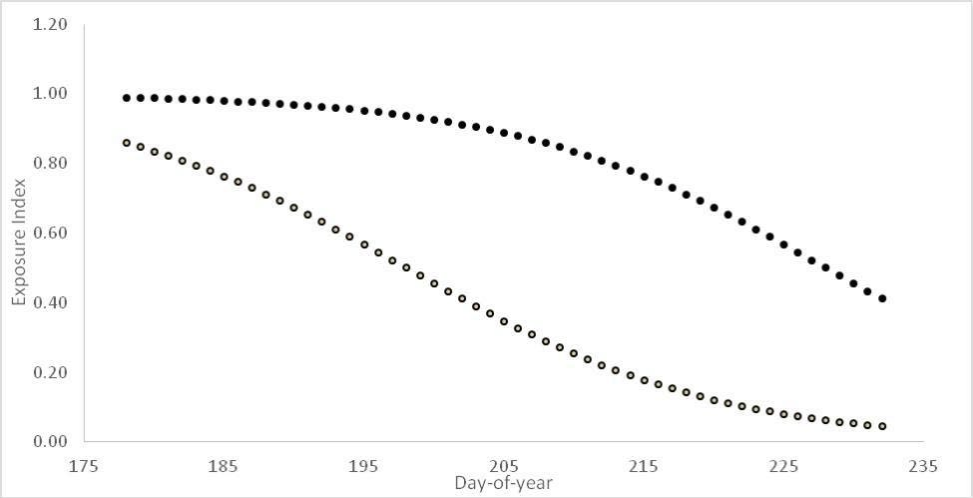
The exposure index of southbound adult (open) and juvenile (filled dots) western sandpipers stopping over on the Fraser River estuary on their respective median passage dates (adult d-o-y 198; juvenile d-o-y 228), in relation to peregrine arrival date. Exposure is higher in years with earlier peregrine arrival, which itself depends on the date of spring onset in coastal Alaska

The exposure index on the median adult passage date using the earliest observed peregrine arrival date (d(*198, 178*) = 0.86) is 17 times higher than with latest recorded arrival date (d(*198, 232*) = 0.05; Fig. 3). Juvenile exposure also climbs with advancing peregrine arrival date. Due to their later passage juvenile exposure is overall higher than that of adults, though the difference between the earliest and latest years is less extreme (0.99 vs. 0.46).

## Discussion

The data and analysis presented here enable quantification of the seasonal pattern of exposure to peregrine falcons of southbound western sandpipers on the Fraser estuary. The data show that the exposure of both adults and juveniles increases as the passage period progresses. The novel result reported here is the large magnitude of the effect of date of peregrine arrival on index values. These observations are consistent with ‘peregrine avoidance’ hypothesis proposed by Lank et al. [14] to explain the early southward migration of western sandpipers.

Though consistent with the hypothesis, these results do not on their own provide a very stringent test. It is possible that early southward migration is the result of other selective factors. For example, timing may be selected to match peak food abundance on stopover sites (previously discussed extensively on p. 321–323 in [14]). A more demanding test is to ask whether migration timing responds to the large variation in the peregrine arrival date. This variation provides a natural experiment because it strongly affects the timing of the seasonal rise in migratory danger.

The benefit of early migratory timing is greater in earlier snowmelt years. Equation (2) reveals, for example, that in an average snowmelt year (*m* = 220), an advance of 5 days from the median adult date (d-o-y 198) would reduce the exposure index from 0.12 to 0.08. The same 5-day advance when *m* = 200 lowers exposure to 0.35 from 0.46. The peregrine avoidance hypothesis thus predicts that in years with earlier spring onset, western sandpipers should respond more strongly, adjusting breeding phenology so that migratory departure from the Arctic is earlier than it otherwise would be.

A critical assumption underlying this prediction is that western sandpipers have the information necessary (i.e. the date of spring arrival) to make the requisite adjustment. This seems reasonable. They arrive in Alaska just a few days after the average snowmelt date and must often experience snowmelt directly. When snowmelt precedes their arrival, the degree of vegetation growth would provide the basis for a good estimate. To test the idea that western sandpipers anticipate migratory predation danger, Hope et al. [28] compared their behavior on the Fraser estuary in two summers with differing peregrine arrival dates (2007 *m* = 214; 2008 *m* = 230). In the more dangerous year (2007), both adults and juveniles had higher vigilance ([28]; see their Fig. 1), fed further from shoreline ([28]; see their Fig. 5), and had greater flight initiation distance in response to experimental walking approaches by a human (see their Fig. 2). These responses were consistent throughout the southward passage period - including the period prior to peregrine arrival. This suggests that sandpipers were generally more cautious throughout 2007, and not merely nervous as a result of a higher encounter rate with peregrines.

The prediction further assumes that advancing migratory departure date is possible at all. Western sandpipers are among the earliest southward migrants, suggesting that the scope to advance the date of southward migration might be limited. Nevertheless, Jamieson et al. [25] found that female western sandpipers shortened the length of the breeding cycle in early snowmelt years. They did so by earlier termination of replacement clutch laying, and by reducing their contribution to parental care, so advancing migratory departure beyond what it otherwise would be by as much as 8 days, with the advance greater when spring is earlier. The net result is that southward passage timing is relatively invariant in spite of large variation in the start of breeding.

Within years, the benefit of an advance in migration is greater on later dates. For example, in an average snowmelt year, a five-day advance from date d-o-y 198 reduces the exposure index from 0.12 to 0.08, but from 0.33 to 0.24 from d-o-y 212. The hypothesis therefore predicts that later-breeders should advance departure more than early breeders. As predicted, later-breeding western sandpiper females give less parental care and depart more quickly thereafter than early breeders [29]. Males provide the bulk of the post-hatching parental care, and do not seem to make similar adjustments. The shortening parental investment of females is manifested as increasingly protogynous (female-first) southward passage on the Fraser estuary [15] in earlier snowmelt years.

Western sandpipers can under some circumstances further mitigate exposure to peregrines with adjustments to migratory speed. (‘Migratory speed’ refers to the rate of geographic progression, including stopover time; [30]). If progressing southward at the same migratory speed as peregrines, Eq. (2) would apply along the entire route, and each migrant would experience the same exposure index at each successive stopover site. But the potential migratory speed of sandpipers (up to ~ 400 km d^− 1^) is greater than of peregrines (~ 200 km d^− 1^; [31]). An increase in migratory speed would enlarge the distance ahead of migratory peregrines, thereby reducing exposure at subsequent stopovers [32].

Higher migratory speed requires more intense feeding [30], attained by foraging with less vigilance, in smaller groups (less competition), or at more dangerous sites where food availability is higher. A moderately large fuel load also enables higher migratory speed. But each of these behavioral adjustments increases vulnerability to predators present at that stopover site. This heightened danger must be weighed against the reduction in exposure to peregrines at subsequent stopover sites. Hope et al. [28, 31] reasoned that in early summer - far ahead of the mid-summer arrival of peregrines - southbound western sandpipers can afford to migrate relatively slowly, maintaining safety from predators resident at stopover sites. As migrant peregrines draw nearer, sandpipers pick up migratory speed in order to maintain a safe distance ahead. But as migrant peregrines begin to arrive and the exposure index rises further, migrants must exercise greater caution. Hope et al. [28] refer to this seasonal adjustment as ‘caution – speed – caution’, and recorded exactly this pattern in the vigilance levels of migrant western sandpipers on the Fraser estuary ([32]; see their Fig. 4). An identical pattern in the length-of-stay was observed on Sidney Island, a nearby stopover site ([31]; see their Fig. 5). Migrants evidently are able to use their internal calendar [33] to adjust migratory behavior in relation to the date-dependent exposure index – in principle even without directly encountering peregrines.

Factors other than predation danger could also affect migratory timing. The literature on shorebirds is largely focussed on seasonal variation in the availability of prey at breeding, stopover or wintering sites (see Discussion in [14]). Western sandpiper adults depart breeding areas while there are 24d of daylight, but juveniles do not depart on their migration until a month later. Food availability on breeding areas therefore remains sufficiently high to support the rapid growth of young birds well beyond the adult departure date. Measures on the Fraser estuary stopover sites show that food availability either increases (invertebrates; see Table 1 in [14]) or remains constant (biofilm; unpubl. data) from July to August. These observations suggest that a seasonal decline food availability is unlikely to underlie early southward migration, at least for adults.

The peregrine avoidance hypothesis holds that timing adjustments keep migratory mortality lower than it otherwise would be. In the terminology used by Creel et al. [34], the ‘inherent danger’ (i.e. the mortality that peregrines and other predators would inflict if sandpipers did not adjust) of southward migration is high, but ‘induced defenses’ (i.e. these and other behavioral adjustments to migration) keep the ‘realized risk’ (i.e. the actual mortality level) lower. As also emphasized by Creel et al. [34], induced defenses have costs, likely paid for in fitness terms in either or both of two main ways. First, shortening the duration of parental care lowers migratory danger for parents, but presumably also lowers the survival chances of offspring. In effect parental safety is purchased at the expense of offspring survival. A second possibility is that some aspect of the condition of migrants is negatively affected by these adjustments in migratory behavior, which is manifested as lowered survival after arrival at non-breeding areas, presumably increasingly so as the adjustments become larger.

## Declarations

### Data availability

The data used here are presented in Fig. 3 of Ydenberg, R.C., R. W. Butler, D. B. Lank, B. D. Smith, and J. Ireland. 2004. Western sandpipers have altered migration tactics as peregrine falcon populations have recovered. Proceedings of the Royal. Society London B 271:1263–1269.More detail, if required, is available from the corresponding author.
